# Lung Tumorigenesis Alters the Expression of Slit2-exon15 Splicing Variants in Tumor Microenvironment

**DOI:** 10.3390/cancers11020166

**Published:** 2019-02-01

**Authors:** Ming-Fang Wu, Cheng-Yen Chuang, Pinpin Lin, Wei-Ting Chen, Shang-Er Su, Chen-Yi Liao, Ming-Shiou Jan, Jinghua Tsai Chang

**Affiliations:** 1 School of Medicine, Chung Shan Medical University, Taichung 40201, Taiwan; mfwu0111@gmail.com; 2 Divisions of Medical Oncology and Pulmonary Medicine, Chung Shan Medical University Hospital, Taichung 40201, Taiwan; 3 Division of Thoracic Surgery, Taichung Veterans General Hospital, Taichung 40705 Taiwan; cychuangtw@gmail.com; 4 National Institute of Environmental Health Sciences, National Health Research Institutes, Zhunan 35053, Taiwan; pplin@nhri.org.tw; 5 Institute of Medicine, Chung Shan Medical University, Taichung 40201, Taiwan; jc610067@gmail.com (W.-T.C.); glucosewater@yahoo.com.tw (S.-E.S.); firmsky02@yahoo.com.tw (C.-Y.L.); 6 Department of Microbiology and Immunology, Chung Shan Medical University, Taichung 40201, Taiwan; 7 Division of Allergy, Immunology and Rheumatology, Department of Internal Medicine, Chung-Shan Medical University Hospital, Taichung 40201, Taiwan

**Keywords:** Slit2 splicing variants, lung cancer, pneumothorax, tumor microenvironment, kRas^G12D^, LPS treatment, inflammation

## Abstract

Slit2 expression is downregulated in various cancers, including lung cancer. We identified two Slit2 splicing variants at exon15—Slit2-WT and Slit2-ΔE15. In the RT-PCR analyses, the Slit2-WT isoform was predominantly expressed in all the lung cancer specimens and in their normal lung counterparts, whereas Slit2-ΔE15 was equivalently or predominantly expressed in 41% of the pneumothorax specimens. A kRas^G12D^ transgenic mice system was used to study the effects of tumorigenesis on the expressions of the Slit2-exon15 isoforms. The results revealed that a kRas^G12D^-induced lung tumor increased the Slit2-WT/Slit2-ΔE15 ratio and total Slit2 expression level. However, the lung tumors generated via a tail vein injection of lung cancer cells decreased the Slit2-WT/Slit2-ΔE15 ratio and total Slit2 expression level. Interestingly, the lipopolysaccharide (LPS)-induced lung inflammation also decreased the Slit2-WT/Slit2-ΔE15 ratio. Since Slit2 functions as an anti-inflammatory factor, the expression of Slit2 increases in kRas^G12D^ lungs, which indicates that Slit2 suppresses immunity during tumorigenesis. However, an injection of lung cancer cells via the tail vein and the LPS-induced lung inflammation both decreased the Slit2 expression. The increased Slit2 in the tumor microenvironment was mostly Slit2-WT, which lacks growth inhibitory activity. Thus, the results of our study suggested that the upregulation of Slit2-WT, but not Slit2-ΔE15, in a cancer microenvironment is an important factor in suppressing immunity while not interfering with cancer growth.

## 1. Introduction

Slit is a secreted glycoprotein, which was first identified as an axon-repellent molecule expressed by the glia cells along the midline of the CNS in *Drosophila*. When bound to a Robo receptor, Slit/Robo complex, it prevents axon from re-crossing the midline [1]. In mammals, three Slit proteins (Slit1, 2, and 3) [2] and four Robo receptors (Robo1, 2, 3, and 4) [3,4,5] have been identified. Slit/Robo signaling plays an important role in the development of multiple tissues, including lung, kidney, heart, and mammary gland [6,7,8,9,10]. The expression of Slit2 is repressed by hypermethylation in many cancers [11,12,13]. In addition to its role in embryogenesis, Slit/Robo signaling studies have focused on its role in tumorigenesis, including cancer cell proliferation, motility, and angiogenesis. However, contradictory roles of Slit/Robo signaling in tumorigenesis have been discovered in various systems [14].

Slit2 has been shown to inhibit the proliferation of gastric cancer cells [15], colorectal carcinoma cells [13], mammary basal cells [7], progenitor cells in the neocortex [16], lymphatic endothelial cells [17], and endothelial cells [18]. In contrast, Slit2/Robo1 signaling plays an oncogenic role in tumorigenesis by enhancing tumor growth in intestinal tumors and osteosarcoma [19,20]. The contradictory roles of Slit2/Robo signaling in tumorigenesis are not well understood and require further investigation.

We identified the exon 15 splicing variants of Slit2—Slit2-WT (containing exon 15) and Slit2-ΔE15 (lacking exon 15). Slit2-WT suppresses lung cancer cell invasion, while Slit2-ΔE15 inhibits both the growth and invasion of lung cancer cells [21]. We also observed that Slit2-ΔE15, but not Slit2-WT, is able to normalize the vessel size [22]. These studies reveal that the Slit2-exon15 splicing forms have differential roles in tumor growth and angiogenesis. The Slit2-WT isoform expression is higher than the Slit2-ΔE15 expression in embryonic lung fibroblast MRC5 cells, while the Slit2-ΔE15 expression is higher than the Slit2-WT expression in normal bronchial epithelial cells (Beas2B) [21]. Thus, we suspect that Slit2-WT and Slit2-ΔE15 play distinct roles in tumorigenesis and normal physiology. This study aims to understand whether tumorigenesis changes the expression ratio of Slit2-WT and Slit2-ΔE15 in a tumor microenvironment.

## 2. Results

### 2.1. Expressions of Slit2-Exon15 Splicing Variants in Lung Cancer and Non-Lung Cancer Patients

**Figure 1:**
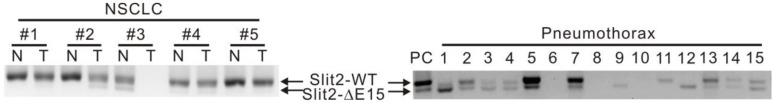
Expressions of Slit2-exon15 isoforms in lung cancer and pneumothorax specimens. Left panel shows the Slit2-exon15 isoforms expressed in the NSCLC (non-small cell lung cancer) specimens. Slit2 is highly repressed in lung tumors. To observe the isoforms in the tumors, the product of two to three PCRs (25 μL/reaction) was precipitated and loaded onto the gel, whereas only 5 μL of PCR was loaded for the normal lung counterparts. Slit2-WT is the predominant form expressed in both normal lungs and lung tumors. Right panel shows the expression pattern of the Slit2-exon15 isoforms in the pneumothorax patients. The expression pattern of the Slit2-exon15 isoforms varies among the specimens.

Slit2 was highly repressed in lung cancer specimens but showed a good expression in adjacent normal lung tissue. We observed that Slit2-WT is predominantly expressed in both lung cancer tissue and adjacent normal lung tissue. Since Slit2-ΔE15, but not Slit-WT, possesses an inhibitive capability in cell proliferation, we wanted to investigate whether the expressions of the Slit2 isoforms are comparable between the normal lung tissue of lung cancer patients and healthy individuals. As it was not possible to obtain normal lung specimens, pneumothorax specimens were used as the nontumor specimens in this study. Interestingly, the Slit2-WT isoform was almost exclusively expressed in all the lung cancer specimens and their normal counterparts, while the Slit2-ΔE15 expression was higher than the Slit2-WT expression in 40.7% of the pneumothorax patients (Figure 1 and Table 1). Due to the low expression level of Slit2 in lung cancer, in order to observe the Slit2 splicing forms in lung tumors, PCR products were precipitated from two to three PCRs and loaded onto the gel for detection. Since the expressions of Slit2-WT and Slit2-ΔE15 were amplified in the same PCR, the amount of PCR product loaded on the gel did not affect their expression ratio. Therefore, there were differential expression patterns of the Slit2 isoforms between the lung cancer and pneumothorax specimens. There are two possible reasons for the predominant expression of the Slit2-WT isoform in the lung cancer specimens: (1) people with the Slit2-WT isoform are at a higher risk of lung cancer, since Slit2-WT lacks the ability to inhibit cell proliferation, and (2) the expression ratio of Slit2-WT/Slit2-ΔE15 changes in response to the development of lung cancer. 

### 2.2. Expressions of Slit2 Isoforms in Normal Lungs and Metastasized Lungs in Nude Mice Injected with Lung Cancer Cells via Tail Vein

**Figure 2:**
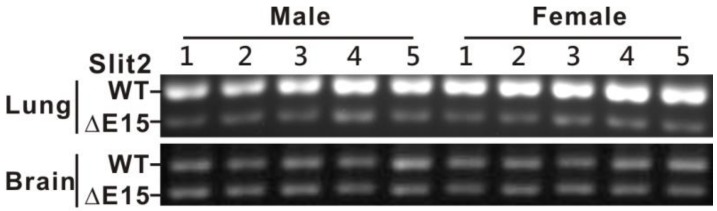
Expressions of Slit2-exon15 isoforms in the lungs and brains of nude mice. In the lungs, there is a higher expression of Slit2-WT than Slit2-ΔE15 in both males and females. In the brain, Slit2-WT and Slit2-ΔE15 are expressed at equivalent levels.

**Figure 3:**
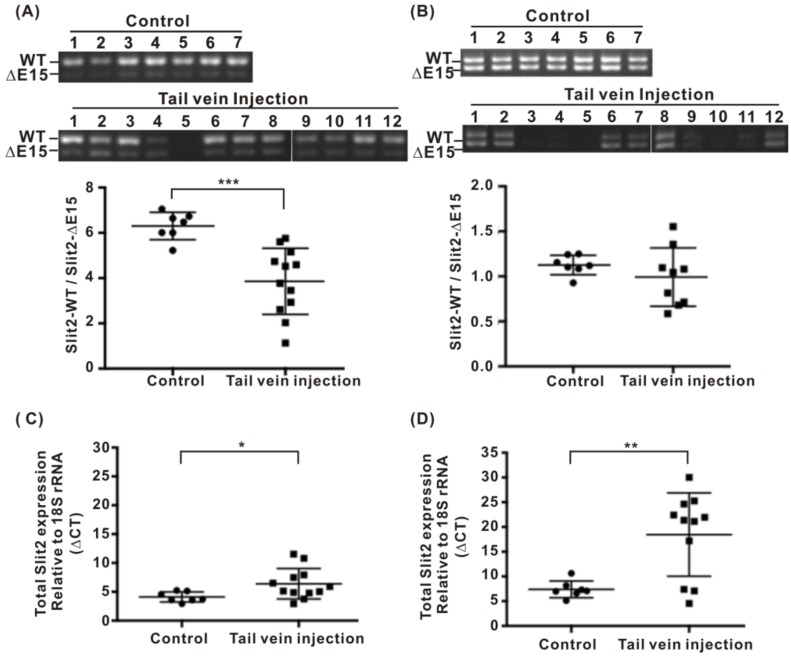
Expression of Slit2-exon15 isoforms in a tail vein injection of lung cancer cells in an animal model. (**A**) Expression of Slit2-exon15 isoforms in the lungs with/without a tail vein injection of lung cancer cells. (**B**) Expression of Slit2-exon15 isoforms in the brain with/without a tail vein injection of lung cancer cells. (**C**) Total expression level of Slit2 in the lungs relative to 18S rRNA. (**D**) Total expression level of Slit2 in the brain relative to 18S rRNA (ΔCT = CT_slit2_ − CT_18SrRNA_; CT: cycle threshold). Higher ΔCT means a lower expression of Slit2. * *p* < 0.05, ** *p* < 0.01, and *** *p* < 0.005.

As there were no normal lung specimens available for detecting the Slit2-exon15 splicing variants and both the normal lung bronchial epithelial Beas2B cells and 40% of the pneumothorax specimens expressed a higher level of Slit2-ΔE15 than Slit2-WT, we investigated whether lung metastasis affects the Slit2 isoform expression in Balb/c nude mice injected with CL1-5 human lung cancer cells into the tail vein. We generated a pair of primers that could distinguish the Slit2-exon15 isoforms in mice but not in humans. Among the control mice, the expression of Slit2-WT was much higher than that of Slit2-ΔE15 in the lungs resulting in a high Slit-WT/Slit2-ΔE15 ratio. Slit2-WT and Slit2-ΔE15 were equivalently expressed in the brain (Figure 2). Among the mice injected with the CL1-5 cells, lung metastasis was significant 45 days after the injection. The ratio of Slit2-WT to Slit2-ΔE15 decreased in the lungs with metastasis compared to the lungs of the control group (Figure 3A) and the overall Slit2 expression was repressed in the lungs of some animals (Figure 3C). The ratio of Slit2-WT to Slit2-ΔE15 did not change significantly in the brain. However, the overall expression of Slit2 was greatly reduced in the brains of mice injected with lung cancer cells (Figure 3D). The reason for the decline in the overall expression of Slit2 in the brain is unclear and further investigation is required. However, we cannot rule out the possibility of brain metastasis by the injection of lung cancer cells. These results indicated that normal mice lungs have a high Slit2-WT/Slit2-ΔE15 ratio and lung metastasis results in a decreased Slit2-WT/Slit2-ΔE15 ratio. However, the decreases in the Slit2-WT/Slit2-ΔE15 ratio were not due to a significantly increased Slit2-ΔE15 level but, rather, to a decreased Slit2-WT level. An injection of lung cancer cells into the tail vein inhibited the overall expression of Slit2 in both the brain and lungs. This observation differed from our expectation in that tumorigenesis shifts the splicing process toward Slit2-WT and not Slit2-ΔE15.

### 2.3. Expressions of Slit2-Exon15 Isoforms in Lung Inflammation Induced by Lipopolysaccharide (LPS)

**Figure 4:**
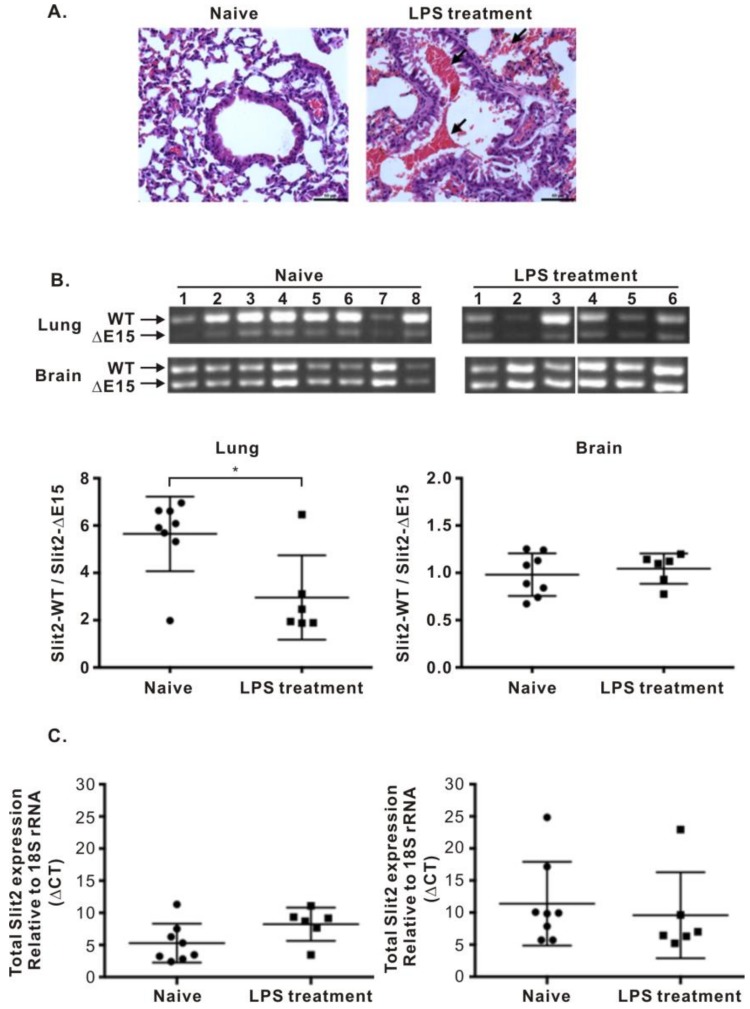
Effects of lipopolysaccharide (LPS) on Slit2 expression. (**A**) Lung inflammation induced by the LPS treatment. (**B**) Expression of the Slit2-exon15 isoforms in the lungs and brains of mice treated with an intranasal administration of LPS. (**C**) Total expression level of Slit2 in the lungs relative to 18S rRNA. (**D**) Total expression level of Slit2 in the brain relative to 18S rRNA. (ΔCT = CT_slit2_ − CT_18SrRNA_; CT: cycle threshold). Higher ΔCT means a lower expression of Slit2. * *p* < 0.05.

The above experiment revealed that the expression of the Slit2-exon15 isoforms and the overall expression of Slit2 can be influenced by lung metastasis. Since inflammation is a risk factor for cancers, we also examined whether lung inflammation affects the expressions of the Slit2-exon15 isoforms. Balb/cByJNarl immunocompetent mice were nasally administered with 10 μg of lipopolysaccharide (LPS) each day for three days and were sacrificed on the fourth day. LPS induced significant inflammation in the lungs (Figure 4A). The expression of the Slit2-WT/Slit2-ΔE15 ratio decreased in the lungs of the LPS-treated group when compared with the naïve group (Figure 4B). The overall Slit2 expression decreased but did not reach statistical significance (Figure 4C). In the brain, the Slit2-WT/Slit2-ΔE15 ratio was unchanged between the naïve and the LPS-treated groups. The overall expression of Slit2 increased in the LPS-treated group but did not reach statistical significance. Thus, LPS-induced acute inflammation in the lungs decreased the expression of the Slit2-WT/Slit2-ΔE15 ratio in the lungs but not in the brain.

### 2.4. Expressions of Slit2-Exon15 Isoforms in kRas^G12D^-Induced Lung Cancer

**Figure 5:**
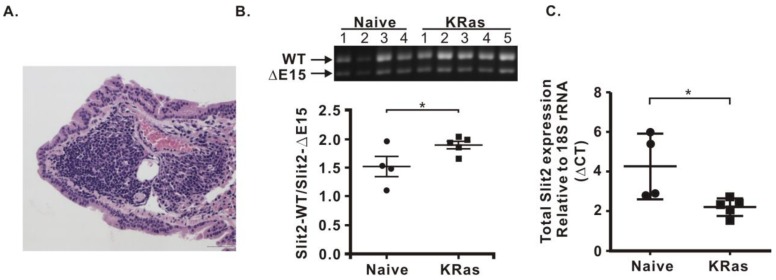
Expressions of Slit2-exon15 isoforms in a kRas^G12D^-induced lung tumor model. (**A**) Representative figure shows that kRas^G12D^ successfully induced lung tumors. (**B**) The ratio of Slit2-WT/Slit2-ΔE15 is higher in the lungs with the kRas induction. (**C**) The expression of the total Slit2 is reduced in the lungs with the kRas induction. (ΔCT = CT_slit2_ − CT_18SrRNA_; CT: cycle threshold). Higher ΔCT means a lower expression of Slit2. * *p* < 0.05.

Although the lung metastasis model via an injection of cancer cells into the tail vein is a common model for studying the implantation of injected cancer cells in the lungs, it does not follow the biological steps for metastasis from a primary tumor. To mimic the development of lung cancer in vivo, we used a classic bi-transgenic inducible system, CCSP-rtTA/Tet-Op-kRas^G12D^, animal model. This model develops adenocarcinomas spontaneously after the administration of doxycycline. The expression of kRas^G12D^ was induced by doxycycline for eight weeks in mice, and the formation of tumors was evident in the lungs of the doxycycline-induced group (Figure 5A). The expression of each Slit2-exon15 isoform was evaluated by PCR and the total expression level of Slit2 was examined by real-time PCR. The results revealed that the Slit2-WT/Slit2-ΔE15 ratio increased in kRas^G12D^-expressed mice, when compared with the naïve group (Figure 5B). In addition, the total expression of Slit2 was greatly elevated in the kRas^G12D^ mice, when compared with the naïve group (Figure 5C). This animal model suggested that the formation of tumors enhances the total Slit2 expression in which Slit2-WT is more highly expressed than Slit2-ΔE15.

## 3. Discussion

Alternative splicing increases the complexity of the coding capacity of the human genome and is implicated in many diseases. The splicing variants of Slit2 at exon 15 revealed distinct functions. Slit2-ΔE15 (absence of exon15) inhibits both the growth and the invasive capability of lung cancer cells, while Slit2-WT (presence of exon15) only inhibits the invasive capability. Slit2 expression is highly repressed in lung cancer specimens when compared with their normal lung counterparts [21]. However, the Slit2-WT splicing form was almost exclusively expressed in both the normal lung and lung cancer specimens in this study. Interestingly, lung bronchial epithelial Beas2B cells express a higher level of the Slit2-ΔE15 splicing form [21]. In the present study, 40.7% of the pneumothorax lung specimens expressed greater or equal amounts of Slit2-ΔE15 when compared with Slit2-WT. Based on the Slit2-exon15 expression patterns in the lung cancer and pneumothorax patients, we proposed two hypotheses to explain why the Slit2-WT isoform is exclusively expressed in lung cancer. First, a high expression level of Slit2-WT is a risk factor for lung cancer, since Slit2-WT does not inhibit cell growth. Second, the tumorigenesis process modulates Slit2-Exon15 splicing toward the Slit2-WT isoform. The first hypothesis is difficult to evaluate, whereas the second hypothesis may be investigated using an animal model.

To address whether lung tumor formation increases the expression of the Slit2-WT isoform, we used a Balb/c nude mice lung metastasis model via a tail vein injection of lung cancer cells. Naïve Balb/c nude mice expressed much higher Slit2-WT than Slit2-ΔE15 in their lungs but equal levels of Slit2-WT and Slit2-ΔE15 in their brain. This revealed that the expression of Slit2-exon15 splicing is regulated in a tissue-specific manner. However, the lung tumors in mice generated via an injection of lung cancer cells into the tail vein showed a reduced expression ratio of Slit2-WT/Slit-ΔE15. This contradicted our expected results. We also observed that the total Slit2 expression level was reduced in the lung tumor group when compared with the naïve group. Interestingly, LPS-induced lung inflammation also reduced the expression ratio of Slit2-WT/Slit2-ΔE15. The total Slit2 expression was lower in the LPS-treated group than in the naïve group but this difference did not reach statistical significance.

The tail vein lung metastasis model revealed that the reduced ratio of Slit2-WT/Slit2-ΔE15 was mostly due to a decrease in Slit2-WT. The change in the Slit2-WT level was also reflected in the reduction of the total Slit2 expression level. This phenomenon is similar to LPS-induced lung inflammation. It is possible that an injection of lung cancer cells into the tail vein induced the lung inflammation. We used a lung kRas^G12D^ animal model to mimic the biological process of initiation and the progression of lung tumors. Interestingly, the expression ratio of Slit2-WT/Slit2-ΔE15 increased in mice with lung tumors when compared with the control, and the total Slit2 expression level also significantly increased in the lung tumor group compared to the control group. The lung kRas^G12D^ model suggested that lung tumorigenesis induces the total expression of Slit2 and mostly the Slit2-WT form, thereby increasing the expression ratio of Slit2-WT/Slit2-ΔE15.

Slit2 has been shown to inhibit the chemokine stromal-derived factor (SDF)-1-mediated leukocyte chemotaxis [23] and the CXCL12/CXCR4-induced chemotaxis of T cells [24], suggesting that it plays an inhibitory role in an immune response. Many studies have demonstrated that Slit2 functions as an anti-inflammatory factor in the regulation of immune responses. During contact hypersensitivity, dendritic cells take up antigens, migrate to lymphoid organs, and become potent antigen-presenting cells to induce an immune response. Slit2 can inhibit dendritic cell migration and suppress the development of a contact hypersensitivity response [25]. Furthermore, Slit2 expression has been found to decrease in a rat glomerulonephritis model, and the inhibition of Slit2 expression in the early stages of the disease has been found to accelerate inflammation [26]. In addition, the expression of Slit2 decreases in the kidneys after ischemia-reperfusion injury (IRI). Administrating Slit2 reduces the tubular injury induced by IRI, presumably through reduced renal neutrophil and macrophage infiltration [27]. Downregulating Slit2 expression may be due to an increase in promoter methylation as a higher Slit2 methylation is associated with an inflammation status in inflammatory bowel disease [28]. Based on its anti-inflammatory role, Slit2 has been implicated as an effective neuroprotector against global cerebral ischemia [29] by stabilizing the blood–brain barrier during a surgical brain injury [30]. It has also been shown to ameliorate renal inflammation and fibrosis in a hypoxic acute kidney injury [31]. N-terminal, but not C-terminal, Slit2 is required to inhibit TGF-β-induced renal fibrosis [32].

An intraperitoneal injection with LPS decreases Slit2 expression in endothelial cells and the liver and enhances endothelial inflammation in mice [33]. In contrast, Slit2 expression is upregulated in an OVA (ovalbumin)-induced allergic airway inflammation in mice [34]. Slit protein plays a role in repulsive axon guidance by its interaction with a Robo receptor, which recruits the Slit2-Robo GTPase-activating protein 1 (srGAP1) to the intracellular CC3 motif of Robo1, subsequently inactivating Cdc42 and reducing actin polymerization [35]. This pathway also functions in the Slit2-mediated anti-inflammatory process, which inhibits immune cell infiltration to the inflammatory sites [26,34,36,37]. However, upregulation of Slit2 in OVA-induced allergic inflammation attracts eosinophils to the airway [34]. Apparently, this is due to a low expression level of srGAP1 in eosinophils, which results in Cdc42 activation and the recruitment of PI3K to Robo1 upon Slit2 stimulation [34]. Thus, Slit2 expression may be regulated differently in response to different pathogeneses of inflammation. In summary, Slit2 plays a role in inhibiting the migration of high srGAP1-expressing leukocytes while inducing the migration of low srGAP1-expressing immune cells.

## 4. Materials and Methods

### 4.1. Study Subjects

In this study, a total of 18 lung cancer and 54 pneumothorax specimens were examined. The lung cancer specimens and normal adjacent tissue were obtained from patients with non-small cell lung cancer who underwent surgical lung resection. The pneumothorax specimens were obtained from the lesion sites of patients with a spontaneous pneumothorax who underwent thoracoscopic surgery. This study was approved by the Institutional Review Board of Chung Shan Medical University Hospital (CSMUH No: CS12153).

### 4.2. Intravenous Injection of Lung Cancer Cells

Four-week-old immunodeficient BALB/CAnN.Cg-Foxn1^nu^/CrlNarl (nude) mice were obtained from the National Laboratory Animal Center (Taiwan) and housed in individually ventilated cages. They were injected with 1 × 10^6^ of CL1-5 lung cancer cells in 100 μL of PBS into the tail vein and sacrificed 45 days later. The lungs and brain were removed from each mouse. The lung tissue of each mouse was divided into two parts. One part was fixed in 10% formalin and the other part was incubated in RNAlater (Thermo Fisher Scientific, Waltham, MA, USA). The brain tissues were incubated in RNAlater only.

### 4.3. Lung Inflammation Induced by Intranasal Administration of Lipopolysaccharide (LPS)

Eight-week-old BALB/cByJNarl mice were obtained from the National Laboratory Animal Center (Taiwan) and housed in individually ventilated cages. These mice were treated intranasally with 20 μl of PBS or 0.5 mg/ml of LPS (Sigma-Aldrich, St Louis, MO, USA), once a day, for 3 consecutive days. They were sacrificed 4 days after the end of the treatment period. The lungs and brain were removed from each mouse for RNA synthesis and part of the lungs was fixed in 10% formalin.

### 4.4. Lung Tumor Formation in kRas^G12D^ Mice

The lung tumor mouse model was generated by Program NP-9 for Translational Innovation of Biopharmaceutical Development-Technology Supporting Platform Axis (Taiwan). All animals in this study were handled in accordance with the guidelines of the Institutional Animal Care and Use Committee (IACUC) of the National Cheng Kung University (NCKU) (Tainan, Taiwan) for the care and use of laboratory animals. Briefly, over 8-week-old FVB-rtTA, Kras2 gene-transgenic mice were administered with doxycycline (2.5 g/L in drinking water) for 8 weeks to induce lung tumor formation. After an additional 4 weeks, the mice were sacrificed and lung tissues were collected.

### 4.5. RNA Extraction and Real-Time PCR

The total RNA of tissues was extracted with TRIZOL reagent (Invitrogen, Thermo Fisher Scientific, Waltham, MA, USA). The total RNA (2 μg) was treated with DNase I and then subjected to cDNA synthesis, according to the manufacturer’s instructions (Promega, Madison, WI, USA). To distinguish the human Slit2-Exon15 isoforms, h-slit2-F (5′-CACCTCTTCGGGCCATT-3′) and h-slit2-R (5′-CAGGGCAAGCCAGATCC-3′) primers were used. To distinguish the murine Slit2-exon15 isoforms, m-slit2-F (5′-TCAGCCCTCAGAGCCATC-3′) and m-slit2-R (5′-ACACTTCTCAGGACAAGCCAA-3′) primers were used. Real-Time PCR was carried out with Q-slit2-F (5′-CCAGAGACCATCACAGAAAT-3′) and Q-slit2-R (5′-GCATCTACCCGAAGGCA-3′) primers using FastStart Universal SYBR Green Master (ROX) (Bio-Rad, Hercules, CA, USA).

### 4.6. Histology

Lung tissues were fixed in 10% formaldehyde, embedded in paraffin blocks, and sectioned at 2 μm. H&E staining was performed using an automated tissue stainer, TST44 (Medite Gmbh, Burgdorf, Germany). 

### 4.7. Statistical Analysis

Pearson chi-square test was performed to compare the Slit2-exon15 isoform expressions between the lung cancer and pneumothorax patients. A two-sided *p*-value <0.05 was considered significant. The differences between the groups were analyzed by a nonpaired *t*-test using GraphPad Prism 6 (GraphPad software Inc., La Jolla CA, USA) and were considered statistically significant at *p*-value <0.05.

## 5. Conclusions

Our studies showed that lung tumors generated by a tail vein injection of lung cancer cells and a LPS-induced lung inflammation decrease Slit2 expression in the lungs, while lung tumors formed in a transgenic Ras^G12D^ animal model increase Slit2 expression, mostly the Slit2-WT form. Thus, Ras^G12D^ transgenic mice-generated lung tumors and tumors generated by an injection of cancer cells into the tail vein possess distinct tumor microenvironments. Lung tumors in Ras^G12D^ transgenic mice increase Slit2 expression, while an injection of lung cancer cells into the tail vein decreases Slit2 expression. Since Slit2 expression is reduced in most inflammatory conditions, this suggests that an injection of lung cancer cells into the tail vein induces lung inflammation. In contrast, Ras^G12D^-induced lung tumors increase Slit2 expression in the lungs, suggesting that Slit2 plays an immunosuppressive role during tumorigenesis. Since the Slit2-ΔE15 splicing form, but not Slit2-WT, is able to inhibit the growth of cancer cells, a higher ratio of Slit2-WT/Slit-ΔE15 expression in the tumor microenvironment is more favorable for the development of lung cancer than a lower ratio of Slit2-WT/Slit-ΔE15 expression. Exon 15 is within the N-terminal of Slit2 and located at the end of the second leucine rich repeat domain (LLR2), which interacts with Robo1 for signaling. Since the N-terminal of Slit2 is responsible for anti-inflammatory activity, it is important to examine whether Slit2-WT and Slit2-ΔE15 have distinct roles in the regulation of the immune system. Thoroughly understanding the biological roles of Slit2-WT and Slit2-ΔE15 in cancer and in an immune response would be helpful for developing therapeutic strategies for inhibiting cancer cell growth and metastasis that do not compromise the immune system.

## Figures and Tables

**Table 1:** Expression of Slit2-exon15 isoforms in lung cancer and pneumothorax patients.

Slit2 Splicing Variants	n	Diseases		*p* ^a^
		Lung Cancer	Pneumothorax	
No. of subjects		18	54	
Slit2-WT ≦ Slit2-ΔE15	22	0 (0.0)	22 (40.7)	0.001
Slit2-WT > Slit2-ΔE15	50	18 (100.0)	32 (59.3)	

**^a^** Data were calculated by using a chi-square test.
